# Soft X-ray Reflection Spectroscopy for Nano-Scaled Layered Structure Materials

**DOI:** 10.1038/s41598-018-34076-5

**Published:** 2018-10-24

**Authors:** A. Majhi, Maheswar Nayak, P. C. Pradhan, E. O. Filatova, A. Sokolov, F. Schäfers

**Affiliations:** 1 Synchrotrons Utilization Section, Raja Ramanna Centre for Advanced Technology, Indore, 452013 India; 2 Homi Bhabha National Institute, Training School Complex, Anushakti Nagar, Mumbai, 400094 India; 3 St Petersburg State University, Ulyanovskaya 3, Peterhof, St Petersburg 198504 Russian Federation; 4 Helmholtz-Zentrum Berlin, Institute for Nanometre Optics and Technology, Berlin, Germany

## Abstract

We introduce a novel approach that addresses the probing of interfacial structural phenomena in layered nano-structured films. The approach combines resonant soft x-ray reflection spectroscopy at grazing incidence near the “critical angle” with angular dependent reflection at energies around the respective absorption edges. Dynamic scattering is considered to determine the effective electron density and hence chemically resolved atomic profile across the structure based on simultaneous data analysis. We demonstrate application of the developed technique on the layered model structure C (20 Å)/B (40 Å)/Si (300 Å)/W (10 Å)/substrate. We precisely quantify atomic migration across the interfaces, a few percent of chemical changes of materials and the presence of impurities from top to the buried interfaces. The results obtained reveal the sensitivity of the approach towards resolving the compositional differences up to a few atomic percent. The developed approach enables the reconstruction of a highly spatio-chemically resolved interfacial map of complex nano-scaled interfaces with technical relevance to many emerging applied research fields.

## Introduction

Today thin films and nano-structured layer systems find a wide range of applications in materials science^1–4^ due to their tunable optical, structural, electronic, magnetic and superconducting properties. Often the quality of films is a governing factor that determines the critical parameters of these devices^5–8^. Any deviation of their physical, chemical and geometrical parameters from desirable ones causes fluctuations in their properties; for example, the complete disappearance of quantum effects in nano-electronic devices or the catastrophic drop of reflectance of ultra short-period x-ray multilayer mirrors. The problem becomes more complicated due to the formation of interlayers, owing to atomic migration, chemical reactions or implantations in metal-oxide-semiconductor gate stacks, which may impact the functionality of the devices by for examples, affecting the effective work function of electrodes^9^, the optical contrast^10^ or their magnetization^11^.

All of these, in turn, stipulate the higher requirements on the technology of thin film synthesis and quality control. One of the key issues is the precise determination of atomic and chemical composition profiles at various interfaces in layered structures with an in-depth resolution approaching the scale of interatomic distances (~1 Å), which pushes the development of novel approaches.

Commonly used transmission electron microscopy (TEM) imaging allows one to attain the desired resolution, provided the interface roughness is small. However, the analysis of quantitative atomic composition appears to be problematic. Additionally, it is a destructive method and the detection of light (low-Z) elements in the presence of high-Z ones becomes difficult^12^. Similarly, the routinely used photoemission spectroscopy or the analysis of the fluorescent radiation in combination with argon ion-sputtering technique are also destructive techniques and may introduce artefacts during sample preparation^13^. Therefore, there is a need for development of alternative analytical techniques (less invasive than TEM), which would allow one to quantify chemically and spatially resolved atomic concentration profiles across the hetero-structure with a depth resolution better than 10 Å. Among the non-destructive techniques, the combined x-ray standing wave and x-ray reflectivity^14^ are limited in their success owing to lack of sensitivity to structures with low contrast interfaces^15^, and/or low-Z materials. Also neutron reflectivity (NR) is complementary to x-ray reflectivity^16,17^, but NR has some known limitations^18^. Finally, hard X-ray photoelectron spectroscopy (HAXPES)^19^ and depth-resolved soft X-ray emission spectroscopy^20^ are the most suitable non-destructive spectroscopic methods. Nevertheless, in all these methods various physical models are used during processing of the original data, which often introduce its own limitations.

In the present work, using resonance phenomena in the soft x-ray region by exploring both spectral and angular dependencies of the reflection coefficient, we develop a novel approach to probe the interfacial structural phenomena in layered nano-structured films. Owing to its excellent chemical sensitivity, high contrast variation and high resolution such a technique could clearly overcome the previously mentioned limitations.

Nowadays spectral dependent reflection spectroscopy emerges as a potential tool for atomic and electronic structures of materials and is utilized in different contexts at relatively larger incidence angles^21–27^. Similarly, reflection spectroscopy at constant momentum transfer q_z_, is utilized in different contexts for epitaxially grown transition-metal oxides^4,28–30^. For example, the sensitivity of constant q_z_-reflection to a marker layer (La_x_Sr_1-x_TiO_3_) with concentration x = 0.006 was demonstrated for a structurally nearly perfect SrTiO_3_ film (ref.^30^). Subsequently, the layered structure was determined by fitting reflection spectra with a-priori information on the film. Similarly, based on the atomic slices approach, reflection spectra of constant q_z_ and angular dependence were modeled for an epitaxial LaSrMnO_4_ film to extract information on layer termination and the stacking sequence of the atomic planes^30^. However, the idea of creating a depth of formation of reflected beam within a nano-scale range near the “critical angle” is not clearly understood. Such an attempt could potentially probe the physico-chemical characteristics of the nano-scaled layer structure by varying the angle of incidence. Additionally, shallow incidence angles near the critical angle provide much more sensitivity. This facilitates the discrimination of the chemical state of the overlaying surfaces from that of the underlying layer. Relying on the above mentioned method, we combine the high sensitivity of energy- and angle-dependent near-edge reflection spectra to obtain a quantitative spectroscopic profile of complex nano-layered structures.

The physics of resonant soft x-ray reflectivity (R-SoXR) is analogous to deuteration in NR, where a tunable contrast enhancement is obtained^31^. The angle-dependent R-SoXR is a layer-specific and element-specific technique, which provides information on (1) structural, electronic and orientational ordering of organic films^18,24,32^, (2) magnetization in magnetic structures^33–35^, (3) ordering in correlated electron systems^36^, and (4) electronic structure and structure of surface layers and thin films^26,27,37–39^. Nevertheless, in case of a more complex interface structure, it is difficult to extract unique real-space information to obtain a chemically resolved atomic distribution profile in a straight-forward manner. Very poor electron density contrast along with different extents of unknown interfacial atomic migration, chemical changes and presence of impurities cause the complex interfacial structures. All these require a proper understanding of the real and imaginary parts of the scattering length density to be modeled. Indeed, here we demonstrate an approach to overcome this difficulty in conjunction with spectral dependent reflection spectra to unambiguously and precisely determine the interfacial profile.

The approach developed combines energy spectra, to identify chemical species by discriminating chemical information of overlaying from that of underlying layers, with angular dependent spectra to quantify chemically- and spatially-resolved atomic profiles. This approach enables to reconstruct a highly precise chemically- and spatially- resolved nano-scaled interfacial map up to tens of nanometre thickness within a few atomic percent in a non-destructive manner. The developed approach is demonstrated on a silicon-boron thin film structure because of its emerging technological interest, where boron is used as a p-type dopant in Si-based microelectronics^40^. However, the approach is applicable to other complex layered structures as well.

## Results

### X-ray reflection spectra calculated near boron K-threshold

**Figure 1:**
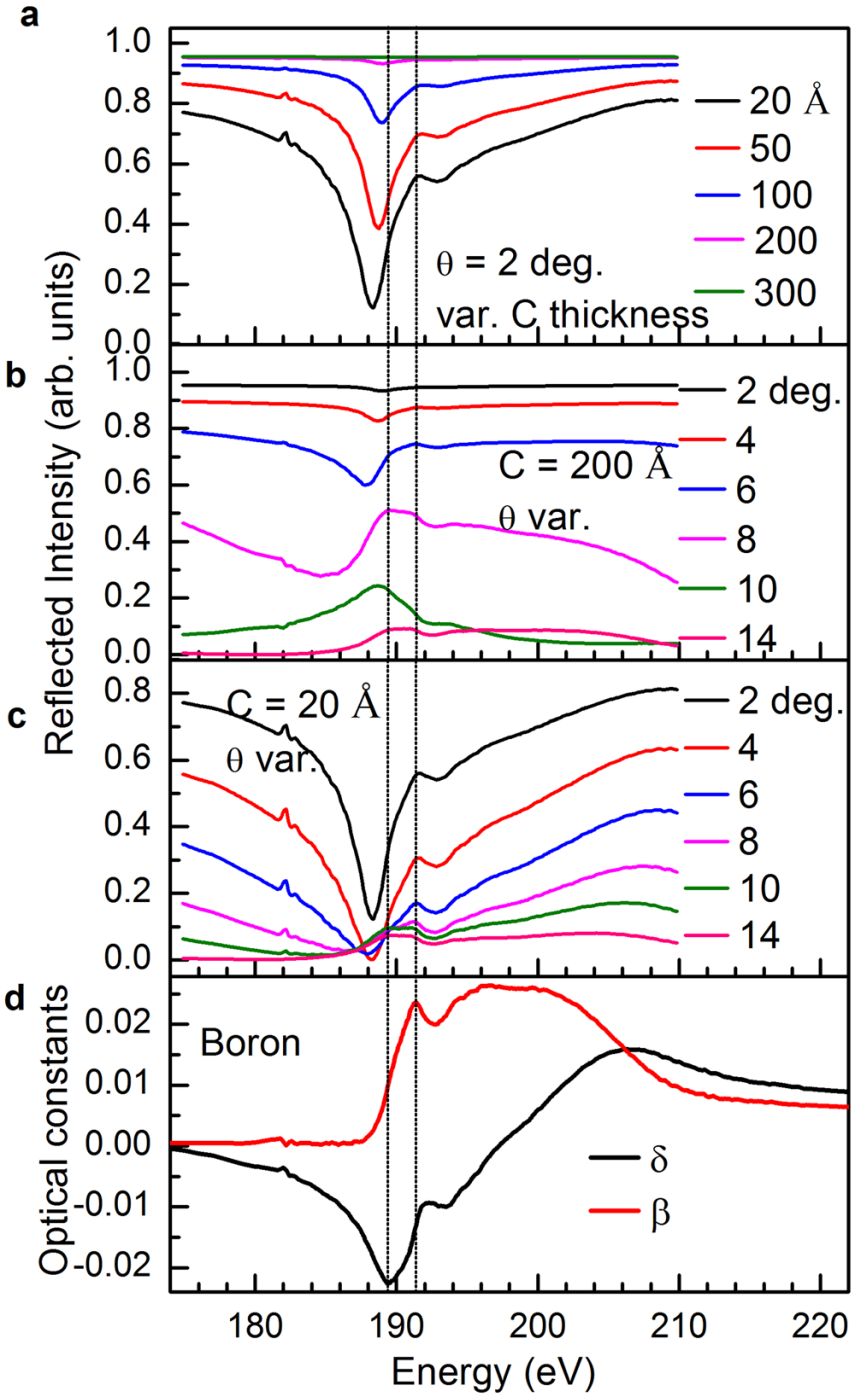
Spectral dependence of the grazing incidence reflection calculated for the B K-edge region along with measured optical constants of elementary boron. The reflection spectra calculated for an ideal structure (described in the text) in Fresnel’s approach (zero roughness and bulk density). (**a**) Reflection spectra at an angle of 2° for different thicknesses of the top C-layer. (**b**,**c**) Reflection spectra for different incidence angles and for a C top layer of 200 Å and 20 Å, respectively. (**d**) Measured near-edge spectral dependencies of optical constants (δ and β) of boron to correlate the features in the spectral dependencies of the reflection coefficient. Dotted vertical lines represent the positions of the minimum (at E ≈ 189.4 eV) in the δ profile and the peak (at E ≈ 191.3 eV) in the β profile.

The calculations of the spectral-dependent reflection in the vicinity of the boron K-threshold (Fig. 1a–c) are done for thin films having layer structure of C (varying thickness)/B (40 Å)/Si (300 Å)/W (10 Å)/oxidized Si substrate. The layer sequence is maintained similar to the fabricated one. For these calculations, measured optical constants of boron (Fig. 1 (d)) are used. The absorption edges of the other elements in the structure (C K-edge, Si L-edge and W N-edge) are far from the boron K-threshold. As a consequence, all the observed fine features in Fig. 1a–c are due to the boron structure. Figure 1a demonstrates tunable sensitivity of the boron layer by varying the thickness of the top carbon layer at a fixed grazing incidence angle of θ = 2°. For the carbon layer, the critical angle for total external reflection (neglecting absorption, ${\theta }_{c}={\sin }^{-1}\sqrt{(2\delta )}$), near B K-edge at 190 eV) is ≈7.35°. However, below *θ*_*c*_, a portion of the electric field penetrates into the material within a few tens of angstrom due to finite absorption in the material. The field penetration depth is also tuned by varying θ near *θ*_c_. For example, the penetration depths for carbon at a photon energy of 190 eV for different incidence angles of 2°, 4°, 6°, 7°, 8° and 15° are 42 Å, 48 Å, 70 Å, 125 Å, 455 Å and 1768 Å, respectively (Henke database)^41^. So, at C layers thickness ≥ 200 Å, the reflected beam is formed predominantly in the carbon layer, hence nearly no structure of boron is observed. However, at C layers thickness ≤100 Å, the structure of the boron layer becomes more and more observable. This confirms the possibility for non-destructive depth-probing of the local atomic structure by spectral-dependent near-edge reflection spectra. Nevertheless, as a result of interference of waves, reflected at the surface and at the interface between carbon and boron, the reflection features can be shifted in energy and their amplitudes can also be changed. This effect can be traced in Fig. 1a,b. Figure 1c reveals that 20 Å of C does not affect the B features any longer. At angles larger than *θ*_*c*_, the contribution from absorption becomes significant and increases with increasing θ. The reflection spectrum at these angles is very similar to the absorption spectrum^42^. Due to the strong absorption of soft x-rays in the material, the dielectric function ε(E) and the refraction index $n(E)=1-d+ib=\sqrt{\varepsilon (E)}$ are complex functions. In this connection, due to a sign-reversal of δ of boron near its K-edge, the condition of total external reflection does not hold here even at extremely low grazing incidence angles. Due to this sign-reversal, the reflection is noticeable and gradually decreases (in a wide angular region), which makes the depth-probing of the local atomic structure possible. The experimental validation is discussed in the next section.

### Experimental results of near-edge reflection spectra

**Figure 2:**
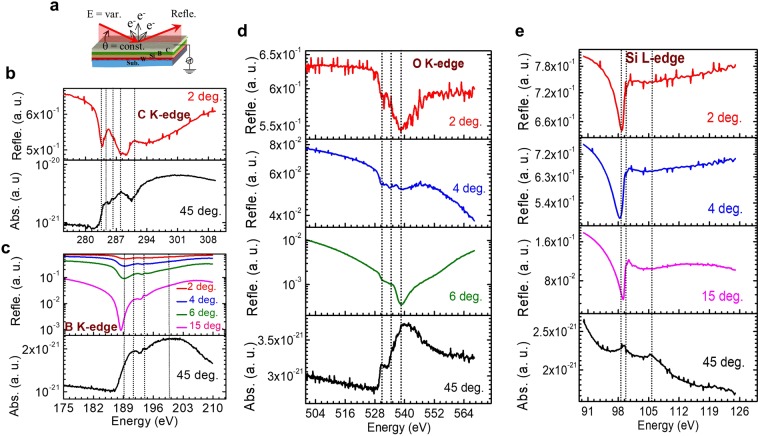
Energy dependence of the measured reflection spectra for different incidence angles in the region of the absorption edges of C, B, O and Si. Measured NEXAFS spectrum at 45 degree near the respective edges is also presented. (**a**) Schematic of measurements. (**b**) C K-edge region. (**c**) B K-edge region at different angles of incidence (**d**) O K-edge region at different angles of incidence. (**e**) Si L-edge region at different angles of incidence. The vertical dotted lines represent the energy positions of different fine structure features as mentioned in the text.

The energy dependence of reflectivity for the system C(20 Å)/B(40 Å)/Si(300 Å)/W(10 Å)/oxidized Si substrate is shown schematically in Fig. 2a. The measurements were made around the respective absorption edges of the elements (colored lines in Fig. 2b–e) at different grazing incidence angles. According to ref.^43^, a qualitative analysis of reflection spectra using localized molecular states formalism without intermediate solution of the Kramers-Kronig equation is possible. Nevertheless, to support the validity of our approach, the reflection spectra are reviewed in parallel with the routinely used near edge x-ray absorption fine structure (NEXAFS) spectroscopy, which were measured simultaneously (black line) at an incidence angle of 45° using the drain current of the sample. Notice that the energetic positions of the C, B, and O K- edges and the Si L- edge differ significantly from each other. It is well known^44^ that the crystal modification of the carbon film grown on top of the substrate depends strongly on the synthesis process (precursors, atoms, or clusters) during synthesis process. The C K-reflection/absorption spectrum (Fig. 2b) reveals that the structure of C originates from both *sp*^2^ and *sp*^3^ states^45^. The first dotted line (≈283.6 eV) is due to the strong C 1 *s* → π* absorption, when reflection intensities undergo a sharp dip. The first peak at ≈284.7 eV (second dotted line) is due to the C 1 *s* → π* transition in the C=C double bond in sp^2^-coordination^44,46^. The second peak at ≈286.1 eV (third dotted line) is related to C 1 *s* → π* transitions induced by the presence of oxygen at the sample surface. The third feature at ≈287.9 eV (fourth dotted line) is assigned to the 1 s → σ*(C-H) transitions^47^. This resonance is attributed to *sp*^3^ coordination. The onset of the smooth edge at ≈291 eV (fifth dotted line) arises from 1 s → σ*(C-C) states. All these features in the reflection spectrum indicating the different chemical environment of carbon are well correlated with the NEXAFS spectrum (black line).

The reflection spectra near the B K- edge (Fig. 2c) was measured at different grazing incidence angles. As discussed in Fig. 1a the structures in Fig. 2c originate predominantly from the boron layer. At small angles (θ = 2°), near-edge features of boron are not clearly observed due to the top carbon layer. As the incidence angle increases, the deeper layers are involved in the reflection process and the local structure from boron becomes more apparent. The minimum in the reflection spectra is ascribed to the sign change of the real part of the atomic scattering factor and hence the real part of optical constant (confirmed from Fig. 1). The contrast changes with incidence angle, which reveal information about the depth dependent chemical states of the layers to discriminate chemical state of the element of the overlaying surfaces from that of the underlying layer. The shape of the B K-edge reflection spectrum correlates well with the absorption spectrum. At the absorption edge (≈189.4 eV), the reflection gets minimum (first dotted line). Li *et al*.^48^ observed that the peak at ≈191.3 eV (second dotted line) is due to the transition of B 1 *s* electrons to unoccupied 2*p*_*z*_ (π*) states; a broad peak (clearly visible in absorption spectrum) in the energy range ≈193 to 207 eV centered at ≈199.8 eV (fourth dotted line) is assigned to the transition of B 1 *s* electrons to unoccupied σ*-states. In both reflection and absorption spectra there is a small feature at ≈193.8 eV (third dotted line). This is due to an overlap of small features of the π -resonance of both the elementary boron and B_4_C (see Fig. 1d and ref.^48^). At this energy the strong π -resonance of B_2_O_3_ is located also^48^. However, the angular reflectivity over wide angles across the strong and sharp π -resonance of B_2_O_3_ indicates the absence of B_2_O_3_ (discussed later).

The reflection data for the O K- edge for different grazing incidence angles (2°–6°) are shown in Fig. 2d. At θ = 4° there is an additional feature near 535 eV. A trace of this feature can be found in the absorption spectrum as well which manifests itself in the asymmetry at the low energy side. This is because, with increasing θ the depth-probing increases, so one can presume that an additional interface is included in the formation of the reflected beam. The analysis of the O K-reflection spectra correlates well with the analysis of the C K-absorption spectrum (discussed earlier) and silicon oxide inside the stack if we assume the top layer is oxidized. Presumably, the main band at ≈538.7 eV (third dotted line) originates from C=O (σ* resonance) and/or from adsorption of water^49,50^ and from transitions of O 1 s electrons to 2p states mixed with Si 3 s, 3p states in SiO_2_. The feature at 531 eV (first dotted line) originates from C=O (π*) bonds^49^. The presence of oxygen is most likely due to adhesion of hydrocarbons and water molecules at the top surface while the sample was exposed to ambient condition before the measurements. Similar features are observed in the absorption spectrum. Notice that contribution of B=O bonds can be ruled out due to the observation of angle dependent reflectivity measurements near B_2_O_3_ edge (B=O often arises due to appearance of B_2_O_3_. See Supplementary Fig. S2 for details).

The reflection spectra near the Si L-edge (Fig. 2e) indicate a pronounced minimum at ≈98.8 eV (first dotted line) which is due to sign-reversal of real part of the optical constant of Si. In accordance with calculated penetration depth at θ = 2°, it is almost impossible to detect a signal from the underlying silicon layer, which is present at 60 Å beneath. However, the observation of a strong silicon signal at θ = 2° indicates that there may be diffusion of silicon into the upper layer. Nevertheless, due to a high sensitivity of the measurement, the real depth may exceed the depth at which the wave field is reduced in e-fold^42^. This means that observation of a signal from a deeper layer may be possible. Further analysis reveal features belonging to Si (99.6 eV) and SiO_2_ (106 eV)^51^. In the next section all this qualitative chemical information obtained so far is used for a quantitative depth- and chemically resolved description of our sample.

### Quantitative spectroscopic depth profile

Prior to the R-SoXR measurements, a microstructural analysis of the sample was done by hard x-ray reflectivity (HXR) measurements at an energy of 8.047 keV (see Fig. S1 in the supplementary material). The best-fit result is obtained by considering Si, B and C as a single layer with a thickness of 352 ± 1 Å, rms roughness of 8.3 ± 0.5 Å and average mass density ≈97 ± 3% of the bulk value of silicon. The equal electron density profiles (EDP) in these three layers reveal that HXR is not sensitive to Si/B and B/C interfaces (see the Supplementary Fig. S1) due to the low electron density contrast^15^ (ideal ${\rm{\Delta }}{\rho }_{B/C}$ ≈ 1.7% and ${\rm{\Delta }}{\rho }_{Si/B}$ ≈ 8.6%). The best-fit result also reveals that the W layer thickness is ≈ 11 Å with a mass density of ≈96 ± 3% of the bulk value with an rms roughness of ≈4 ± 0.5 Å. The rms roughness of the Si substrate is ≈4.5 ± 0.5 Å. A native oxide at the top of the Si substrate is also considered with a thickness of ≈15 Å and an rms roughness of ≈3.5 ± 0.5 Å. Apart from inability to probe C/B and B/Si interfaces due to too low contrast, HXR is also not suitable to provide information concerning interfacial atomic diffusion/chemical changes in the layers and/or contamination due to the presence of impurity in the layers.

**Figure 3:**
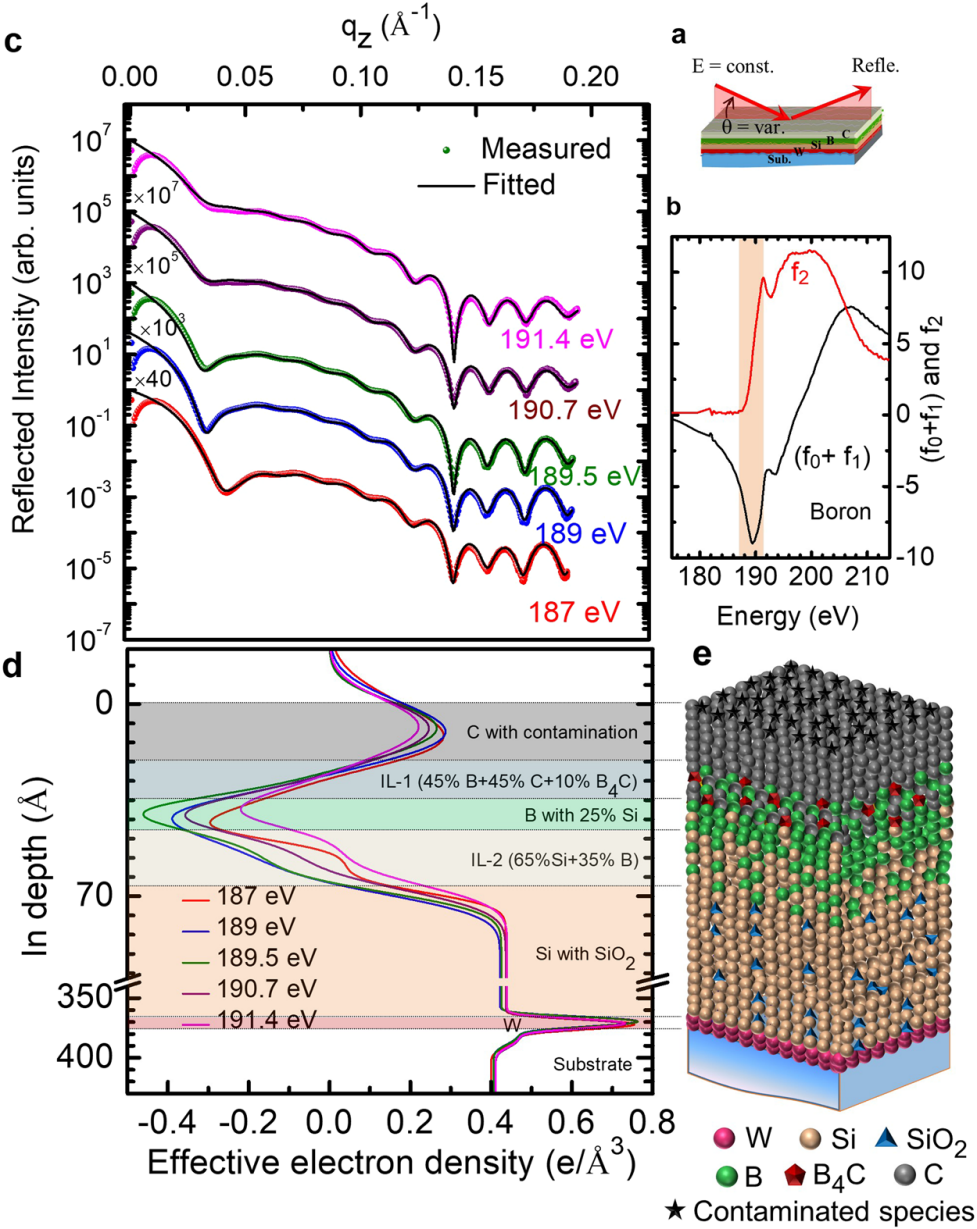
Quantitative structural and compositional analysis using angular resolved R-SoXR around the B K-edge. (**a**) Schematic of angular reflectivity measurements. (**b**) Atomic scattering factors of boron near the B K-edge. The shadowed region indicates the spectral range over which the R-SoXR measurements were performed. (**c**) The measured and fitted R-SoXR spectra at the selected energies. The spectra are shifted vertically for clarity. (**d**) The depth-dependent effective electron density profiles obtained from the best-fit R-SoXR results. (**e**) Schematic model used for the best-fit R-SoXR data to obtain the spatial composition.

To quantify the chemically resolved depth distribution profile, the angular dependence of reflected x-rays was measured at selected energies below, above and close to the respective absorption edges of the elements. Figure 3c shows the measured R-SoXR curves in the vicinity of the B K-edge of elementary boron at the selected energies (187 to 191.4 eV). These curves hold information about the chemical state and the nature of the spatial variation of resonating boron atoms in the layered structure. In this narrow energy range (shadowed region in Fig. 3b) the atomic scattering factor of the elementary boron undergoes a strong variation, whereas all other elements (for e.g., Si, C and W) present in the structure exhibit a flat optical response. The oscillation pattern of R-SoXR curves get strongly modulated due to the interference of reflected amplitudes from the surface and different interfaces constituting the system. This observation clearly corroborates that in contrast to HXR R-SoXR is sensitive to low contrast C/B and B/Si interfaces. Additionally, to get information about the chemical state of boron atoms in the system, the R-SoXR measurements were performed near the B K-edge of B_2_O_3_ (194.1 eV). They exhibit similar behavior with each other near the B K-edge of B_2_O_3_ even the atomic scattering factor of B_2_O_3_ shows a strong variation (see Supplementary Fig. S2). This indicates no boron oxide formation within the experimental limit due to the presence of the carbon cap layer.

The distribution of elements and their chemical nature in a layer structure is determined unambiguously using an approach based on simultaneous data fitting^38,39^. It is also briefly explained in the methods section. To obtain consistent and reliable structural as well as compositional parameters, measured R-SoXR data at the selected five different energies near the B K-edge of elementary boron are fitted simultaneously (Fig. 3c). The details of the fitting procedure, particularly the influence of the fit parameters to the quality of the fit are described in the supplementary material (Supplementary Fig. S3). Even a 5% variation of resonating boron atoms from that of the best-fit value move the fitted profile significantly away from the measured curve due to the strong variation of optical properties of the layer. For example, at 190 eV, by adding 5% boron atoms into the carbon layer *δ* changes from 8.13 × 10^−3^ to 6.79 × 10^−3^ and β changes from 6.57 × 10^−4^ to 1.64 × 10^−3^. So, the scattering contrast at the interface, ${({\rm{\Delta }}\delta )}^{2}+{({\rm{\Delta }}\beta )}^{2}$, which is proportional to scattering intensity, undergoes a significant and tunable enhancement.

Effective EDPs obtained from the best-fit results undergo a strong variation at the interfaces indicating strong sensitivity to C/B and B/Si interfaces (Fig. 3d). The effective EDP of the layers (B layer as well as interlayer), containing the resonating boron atoms, undergoes significant variation as the energy is changed near the elementary boron K-edge. It is clear that the boron layer is in the middle of silicon and carbon layers where the effective EDP value is negative due to a sign-reversal of the real part of the optical constant. The best-fit result clearly indicates that the C/B and B/Si interfaces are not sharp. There is a mixed interlayer at the both C/B (IL-1) and B/Si (IL-2) interfaces. The best-fit results of the thickness, rms roughness and composition of layers obtained using R-SoXR are shown in Table 1. The IL-2 is thicker than IL-1 due to more atomic diffusion at the former interface. The top carbon layer has an average low effective electron density of ≈0.364 e/Å^3^ (bulk value = 0.429 e/Å^3^) due to contamination with hydrocarbon and other contaminants (as observed using reflection/absorption spectroscopy) while the sample was exposed to ambient conditions. The optical properties of the carbon layer do not change significantly near the B K-edge indicating the absence of boron in this layer. The truncation in the effective EDP of the C layer (Fig. 3d) is due to the combined effect of a relatively smaller C layer thickness, a large negative effective EDP value of the underlying layer and the roughness. The observation of the B_4_C signal is due to the formation of C-B bonds which originates mostly from the mixing of boron and carbon atoms in the IL-1 at C/B interface. Furthermore, the best-fit results show that the boron layer is not pure. 25 ± 3% of silicon atoms have diffused into the upper boron layer. Similarly, the best-fit results also reveal that the interlayer IL-2 is composed of 65 ± 3% silicon and 35 ± 3% boron. The system under consideration is characterized by formation of an film by Volmer-Weber growth mode, which in turn leads to the formation of the interface layer with stoichiometry B:Si ≈1:2, where further silicon clusters or a solid solution of Si and B is formed. Only after these steps the growth of boron film begins. In Fig. 3d, the effective electron density of the silicon layer is slightly higher than that of pure silicon (see effective EDP of Si-substrate). This is due to the presence of SiO_2_ which has a higher effective EDP. The partial contamination of deposited Si with oxygen may arise during fabrication of the samples. The sensitivity of R-SoXR for oxide contamination of Si in other layers containing both boron and silicon (e.g., IL-2 and the boron layer with 25% of Si) is negligible near the B K-edge, where boron exhibits a large variation of optical properties, whereas both Si and silicon dioxide exhibit nearly a flat optical behavior. Therefore, based on R-SoXR data, silicon dioxide in the IL-2 and the boron layer are not shown in the schematic Fig. 3e. The amount of oxide contamination of silicon can be quantified by analyzing the R-SoXR data in a similar manner near the Si L-edge. A schematic model of the in-depth atomic distribution within different layers obtained from the best-fit R-SoXR results is shown in Fig. 3e. Thus, R-SoXR precisely detects and quantifies atomic migration across the interfaces, a few percent of chemical changes and the presence of impurities from the top surface to buried interfaces although the electron density difference between the elements is very small.

**Table 1:** The best-fit results of the thickness, rms roughness and composition of the layers obtained using R-SoXR.

Layer	Thickness (Å)	Roughness (Å)	Composition (±3%)
Carbon	21 ± 2	10 ± 2	C with contamination
IL-1	15 ± 1	8 ± 2	45% B + 45% C + 10% B_4_C
Boron	9 ± 1	5 ± 1	75% B + 25% Si
IL-2	22 ± 3	8 ± 2	65% Si + 35% B
Silicon	298 ± 3	4 ± 2	Si with SiO_2_
W	8 ± 1	2.7 ± 0.5	W

## Conclusion

In conclusion, an effective approach is developed to determine the element-specific depth-distribution concentration profile and the chemical state of a nano structured sample in non-destructive manner using resonant reflection spectroscopy in the soft x-ray region. While grazing incidence reflection spectra discriminate the element-specific chemical state of the overlaying from that of underlying surfaces, the angular resonant curves precisely quantify its depth profile in a nano structured configuration. The novel approach presented in this report ultimately enables to reconstruct an interfacial map of element specific, spatio-chemically resolved atomic profile from the free surface to deeply embedded layers. The present study will stimulate activities for other low-Z systems and could open a path for further advancement of resonant soft x-ray reflection spectroscopy to reconstruct structural/chemical interfacial maps. This will also yield considerable progress in the fundamental understanding of more complex functional nano structured materials relevant to number of scientific and technological applications in materials science.

## Methods

Thin film samples are fabricated on ultrasonically cleaned oxidized silicon wafers using electron beam evaporation system with a base pressure of ≈3 × 10^−8^ mbar. A W layer of 10 Å is deposited on the substrate prior to the silicon layer of thickness ≈300 Å to provide optical contrast between the substrate and deposited silicon. Then a boron layer of ≈40 Å is deposited on the silicon followed by a carbon capping layer of thickness ≈20 Å to prevent the formation of native oxide. The deposited thickness is monitored by a quartz crystal using Inficon IC-5 controller and deposition rate is maintained at ≈12 Å/min. HXR measurements are done using a Bruker D8-Discover system at Cu-K_α_ wavelength. Soft x-ray resonant reflectivity measurements are done in s-polarization geometry using the Optics Beamline^52^ at the BESSY-II storage ring. Near the B K-edge, the energy resolution of the beamline is E/ΔE ≈ 2000. For soft x-ray angular measurements, the data are collected up to theta = 89.2°. A GaAsP-photodiode of 4 × 4 mm^2^ acceptance area at a distance of 310 mm from the sample is used. The x-ray absorption data are collected simultaneously along with spectral dependent reflectivity. Absorption data are measured in total electron yield mode by measuring the drain current from the sample which is suitably isolated from the ground. The measured data are accurately normalized to the beam flux.

R-SoXR data are fitted using Parratt formalism^53^. The analysis of R-SoXR data requires a precise value of the optical constants, δ and β (refractive index n = 1 − δ + iβ) of materials near the absorption edge, which is related to the atomic scattering factor, $f(E)={f}_{0}+{f}_{1}(E)-i{f}_{2}(E)$. The lack of fine structure features and the uncertainty of the optical data tables Henke database^41^ in the near edge region due to atomic like assumptions requires to precisely measure the optical constants for an R-SoXR analysis. We obtained more accurate optical constants near the K- edge of boron for elementary B, B_4_C and B_2_O_3_ using the measured absorption data^48^ and using Kramers-Kronig relation^21^. The energy dependent reflection spectra near the B K-edge are calculated by IMD software^54^ using the measured optical constants of boron. During modeling of the R-SoXR measured data, the starting values for rms roughness of the substrate, W and the top surface; densities of W and single layer (Si + B + C); and thicknesses of W and the total film is obtained from the HXR-data. Again, starting guess for thicknesses of Si, B and C is used as per deposited values. The optical constants of W, Si, C, SiO_2_ (non-resonating materials near B K-edge) are taken from Henke *et al*.^41^. The mass densities used for calculation of optical constants of B, C, B_4_C, B_2_O_3_, Si, SiO_2_ and W are 2.34, 2.2, 2.52, 2.46, 2.33, 2.2 and 19.3 g/cc, respectively. The modeling of the R-SoXR data is performed by slicing to different layer structures through different iterations by considering the starting guess of initial deposition sequences of the layers, the chemical information obtained from near-edge reflection spectra. The different iterations are performed by simultaneous fitting of the measured data at different energies. This approach provides the structural parameters and the chemically resolved atomic profile unambiguously.

## Electronic supplementary material

Supplementary materials
