# Supplementary material for: Soft X-ray Reflection Spectroscopy for Nano-Scaled Layered Structure Materials
Source: Sci Rep. 2018 Oct 24;8:15724. doi: 10.1038/s41598-018-34076-5 (PMC6200723; doi:10.1038/s41598-018-34076-5)
Supplement: Supplementary file 1 — Supplementary materials [file 41598_2018_34076_MOESM1_ESM.pdf]

Soft X-ray Reflection Spectroscopy for Nano-Scaled Layered Structure Materials

A. Majhi, Maheswar Nayak, P. C. Pradhan, E. O. Filatova, A. Sokolov and F. Schäfers

Supplementary Materials

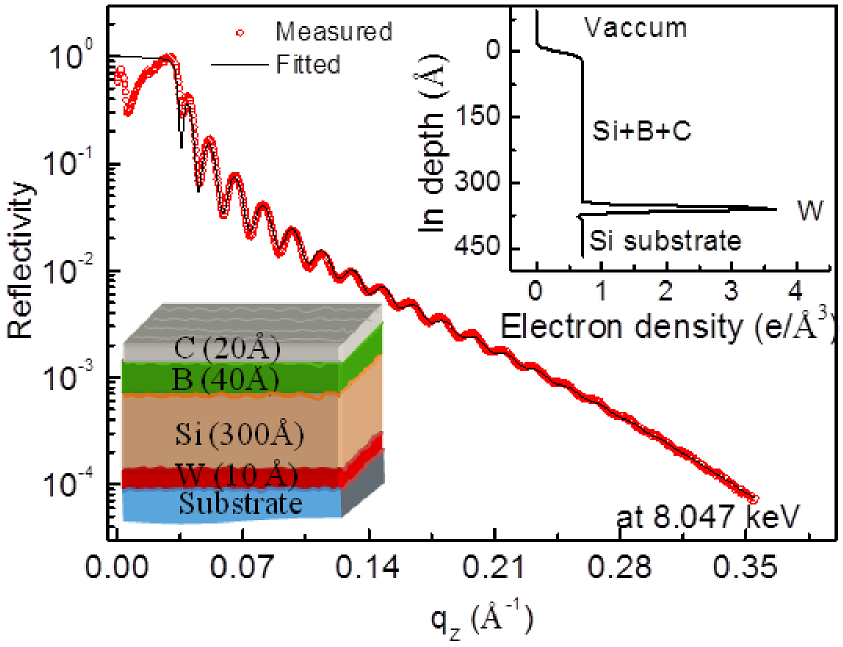

Supplementary Figure S1 | Measured and fitted Hard X-ray reflectivity (using Cu K_α source, E=8.047 keV) curves of the sample shown schematically in the inset. A carbon capping layer is deposited on the top of the sample to avoid the oxidation of the boron layer. The top inset shows the electron density profile (EDP) obtained from the best-fit HXR result. The best-fit result is obtained by considering Si, B and C as a single layer. No variations of EDP in these three layers, indicating that HXR is not sensitive to the Si/B and B/C interfaces due to the low electron density contrast (ideal $\Delta\rho_{B/C}$ ≈1.7% and $\Delta\rho_{Si/B}$ ≈8.6 %).

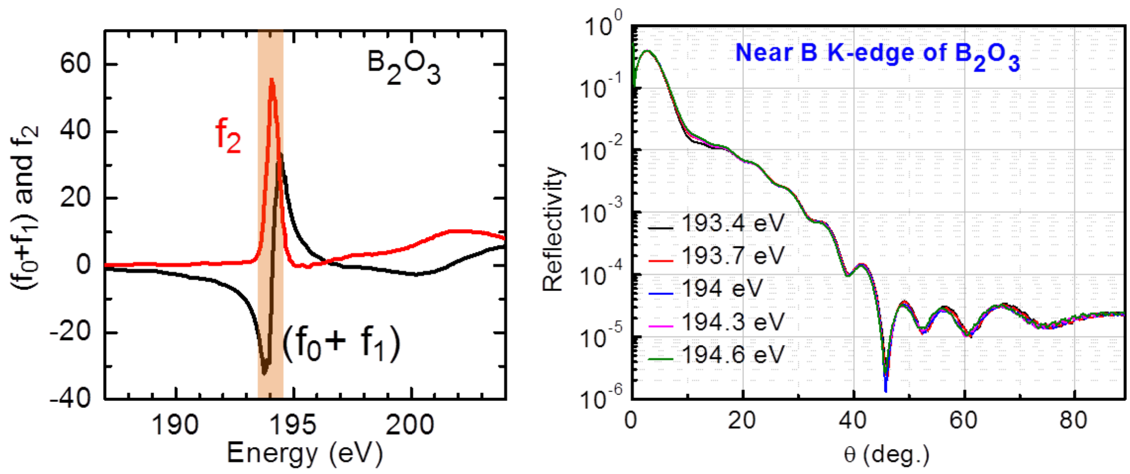

Supplementary Figure S2 | Measured R-SoXR profiles at the selected energies near the B K-edge of B_2O_3. The R-SoXR profiles (at the right hand side) show nearly an identical feature as the energy is tuned near the B K-edge of B_2O_3 although atomic scattering factor of B_2O_3 has a strong variation as shown in the figure at the left hand side. The shadowed region indicates the energy range over which the measured R-SoXR profiles were presented. This indicates that the absence of B_2O_3 in the film within the detection limit. The absence of B_2O_3 may be due to the presence of the carbon cap layer at the top which prevents oxidation of the boron layer when the sample was exposed to the ambient condition.

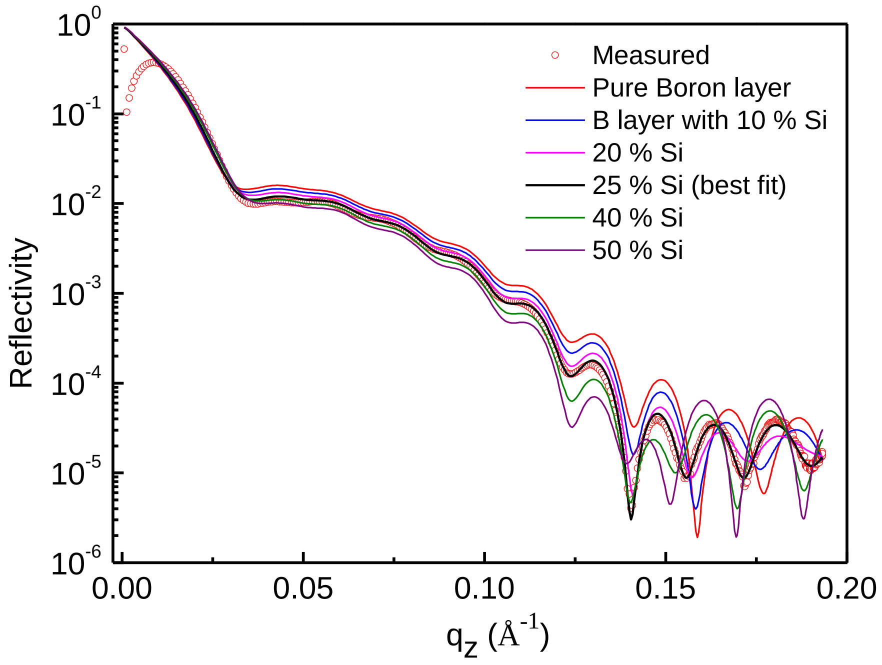

Supplementary Figure S3 |The measured and the fitted R-SoXR data at 190.7 eV demonstrating the sensitive of R-SoXR to atomic composition of the resonating atoms in a layered structure. All the structural parameters which were obtained from the simultaneous data fitting at different energies are kept fixed. Only the composition of the middle boron layer is varied by incorporating different percents of silicon into boron layer. As the atomic percent of boron varies in the middle boron layer, the reflectivity profiles undergo a strong modulation. The best-fit results show the presence of 25 percent of silicon in the middle boron layer. This may be due to diffusion of the silicon into the upper boron layer. Even a 5 % variation of resonating boron atoms from that of the best-fit value move the fitted profile significantly away from the measured curve. This indicates a high sensitive of R-SoXR for compositional analysis within a few atomic percent.
